# Serum Tau Proteins as Potential Biomarkers for the Assessment of Alzheimer’s Disease Progression

**DOI:** 10.3390/ijms21145007

**Published:** 2020-07-15

**Authors:** Eunjoo Nam, Yeong-Bae Lee, Cheil Moon, Keun-A Chang

**Affiliations:** 1 Department of Pharmacology, College of Medicine, Gachon University, Incheon 21936, Korea; ej0817@hanmail.net; 2 Neuroscience Research Institute, Gachon University, Incheon 21565, Korea; yeongbaelee@gachon.ac.kr; 3 Department of Neurology, Gil Medical Center, Gachon University, Incheon 21565, Korea; 4 Department of Brain Science, Graduate School, Daegu Gyeungbuk Institute of Science and Technology, Daegu 42988, Korea; cmoon@dgist.ac.kr; 5 Department of Health Sciences and Technology, GAIHST, Gachon University, Incheon 21936, Korea

**Keywords:** tau protein, serum, Alzheimer’s disease, biomarker, exosome

## Abstract

Total tau (t-tau) and phosphorylated tau (p-tau) protein elevations in cerebrospinal fluid (CFS) are well-established hallmarks of Alzheimer’s disease (AD), while the associations of serum t-tau and p-tau levels with AD have been inconsistent across studies. To identify more accessible non-invasive AD biomarkers, we measured serum tau proteins and associations with cognitive function in age-matched controls (AMC, *n* = 26), mild cognitive impairment group (MCI, *n* = 30), and mild-AD group (*n* = 20) according to the Mini-mental State Examination (MMSE), Clinical Dementia Rating (CDR), and Global Deterioration Scale (GDS) scores. Serum t-tau, but not p-tau, was significantly higher in the mild-AD group than AMC subjects (*p* < 0.05), and there were significant correlations of serum t-tau with MMSE and GDS scores. Receiver operating characteristic (ROC) analysis distinguished mild-AD from AMC subjects with moderate sensitivity and specificity (AUC = 0.675). We speculated that tau proteins in neuronal cell-derived exosomes (NEX) isolated from serum would be more strongly associated with brain tau levels and disease characteristics, as these exosomes can penetrate the blood-brain barrier. Indeed, ELISA and Western blotting indicated that both NEX t-tau and p-tau (S202) were significantly higher in the mild-AD group compared to AMC (*p* < 0.05) and MCI groups (*p* < 0.01). In contrast, serum amyloid β (Aβ_1–42_) was lower in the mild-AD group compared to MCI groups (*p* < 0.001). During the 4-year follow-up, NEX t-tau and p-tau (S202) levels were correlated with the changes in GDS and MMSE scores. In JNPL3 transgenic (Tg) mice expressing a human tau mutation, t-tau and p-tau expression levels in NEX increased with neuropathological progression, and NEX tau was correlated with tau in brain tissue exosomes (tEX), suggesting that tau proteins reach the circulation via exosomes. Taken together, our data suggest that serum tau proteins, especially NEX tau proteins, are useful biomarkers for monitoring AD progression.

## 1. Introduction

Alzheimer’s disease (AD) is the most common neurodegenerative disorder, currently afflicting over 35.6 million individuals worldwide [1,2]. The disease is characterized behaviorally by progressive dementia and pathologically by local accumulations of amyloid β (Aβ) peptide and neurofibrillary tangles (NFTs) composed of tau protein in the brain [3]. Both Aβ accumulation and aggregation of tau in NFTs are believed to contribute directly to AD neurodegeneration and the associated cognitive deterioration [4]. Tau is a microtubule-associated protein that serves to stabilize axonal microtubule bundles, which are essential structural elements in the axonal cytoskeleton [5]. Tau expression is abundant in the central nervous system, particularly in distal axons. Tau contains multiple phosphorylation sites, and phosphorylation status influences microtubule stability, distribution, and function in neurons [6]. If the tau protein is phosphorylated excessively (hyper-phosphorylated), it detaches from the microtubule, resulting in destabilization and disruption of axonal morphology and dynamic transport function [7,8].

At present, a definitive AD diagnosis is possible only by postmortem verification of Aβ deposits (plaques) and NFTs, thus clinicians rely on clinical diagnostic criteria, such as the National Institute of Neurological and Communicative Disorders and Stroke-Alzheimer’s Disease and Related Disorders Association (NINCDS-ADRDA) guidelines, the Diagnostic and Statistical Manual of Mental Disorders, 4th Edition (DSM-IV), and the 10th revision of the International Statistical Classification of Diseases and Related Health Problems (ICD-10); however, the diagnostic accuracy of these AD criteria is poor [9,10]. Therefore, reliable biomarkers capable of detecting AD pathologies are required. Currently, available biomarkers for AD are based on either cerebrospinal fluid (CSF) or neuroimaging. According to a 2016 meta-analysis of fluid AD biomarkers [11], CSF Aβ_1–42_, total tau (t-tau), and phosphorylated tau (p-tau) can distinguish AD patients from controls [12]. Indeed, elevated CSF levels of t-tau, p-tau, and a decreased level of the Aβ_1-42_ are a core sign of AD [12]. However, CSF sampling and analysis are invasive, painful, time-consuming, and expensive [13,14]. Blood is, therefore, a more desirable target for AD biomarker analyses, and several authors have investigated t-tau and p-tau protein levels in the blood of AD patients, but the results are controversial [15,16]. A few previous studies have reported that plasma tau reflects brain tau levels [17,18] and that plasma tau levels are specifically elevated in AD patients [19]. Further, another study reported an association between plasma tau level and CSF t-tau or p-tau (T181) level [20], suggesting the utility of serum tau measures for AD diagnosis. A recent study also reported that t-tau/Aβ_1-42_ in plasma was highly predictive of brain tau deposition and associated with the longitudinal changes in cerebral amyloid deposition, brain glucose metabolism, and hippocampal volume change [21]. However, variation was large across the few available studies on AD serum markers, necessitating further examination for diagnostic reliability and association with clinical features such as cognitive dysfunction.

Exosomes (EX) are endosome-derived membrane vesicles containing proteins and other constituents of cellular origin that act as biological barrier-permeable carriers for local endocrine signaling [22,23]. Exosomes found in blood demonstrate protein expression profiles that reflect pathological changes within disease-specific brain regions, suggesting that analysis of circulating EX protein expression may be an effective non-invasive strategy for monitoring brain pathogenesis [24,25]. For instance, one study reported that levels of p-tau in brain-derived blood EX predicted the development of AD before clinical onset [26], but no subsequent study has examined if EX protein expression can reflect AD severity.

Therefore, in this study, we measured serum tau proteins, including tau proteins within the circulating EX fraction, in AD patients and age-matched controls at baseline and over disease progression to evaluate both the diagnostic efficacy of tau and the association with the severity of cognitive dysfunction. In addition, we measured EX tau in both the serum and brain tissue of JNPL3 mice expressing mutant human tau mutation (MAPT) to verify the EX-mediated transport of brain pathogenic proteins into the blood.

## 2. Results

### 2.1. Serum t-tau and p-tau Protein Levels in Controls, Mild Cognitive Impairments, and Mild-AD Patients

Table 1 summarizes the clinical and demographic characteristics of the study population. We divided subjects into an age-matched control group (AMC, *n* = 26), a mild cognitive impairment (MCI) group (*n* = 30), and a mild-AD (Mild-AD) group (*n* = 20) according to neurocognitive test scores. We compared the average age of the group to exclude the effects of age, one of risk factor in AD, and it showed similar distribution among the groups (AMC, 73.92 ± 0.88 years; MCI, 75.13 ± 0.99 years; Mild-AD, 76.55 ± 1.33 years). As shown in Table 1, disease severity was significantly greater in the Mild-AD group compared to the MCI group as indicated by the significantly lower MMSE scores (AMC, 27.69 ± 0.16; MCI, 23.17 ± 0.20; Mild-AD, 16.55 ± 0.52, *p* < 0.01 vs. MCI), higher CDR-SOB scores (AMC, 0.75 ± 0.07; MCI, 2.55 ± 0.03; Mild-AD, 4.48 ± 0.28, *p* < 0.1 vs. MCI), and higher GDS scores (AMC, 2.00 ± 0.00; MCI, 3.00 ± 0.00; Mild-AD, 3.75 ± 0.14).

**Figure 1:**
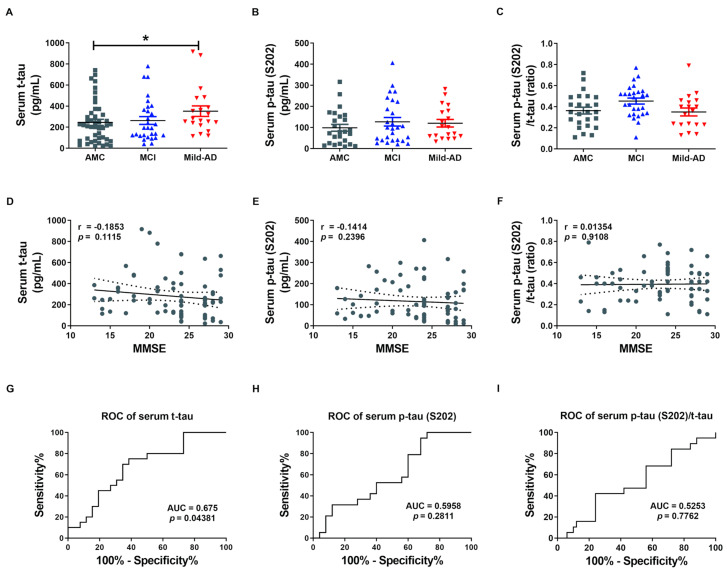
Elevated serum total tau (t-tau) protein in patients with mild Alzheimer’s disease (AD). (**A**) Total tau (t-tau), (**B**) phosphorylated (p)-tau (S202), and (**C**) p-tau (S202)/t-tau ratio in human serum were quantified using ELISA. Serum t-tau was higher in the Mild-AD group compared to the age-matched control (AMC) group. All data were shown as means ± SEM. * *p* < 0.05 compared to the AMC group by one-way ANOVA and post hoc Dunn’s multiple comparison test. Correlations of serum (**D**) t-tau, (**E**) p-tau (S202), and (**F**) p-tau (S202)/t-tau with Mini-mental state examination (MMSE) scores were assessed by the nonparametric Spearman’s rank correlation test. Graphs show regression lines with 95% confidence intervals. Serum t-tau was significantly correlated with MMSE scores. Receiver operating characteristic (ROC) analyses of serum (**G**) t-tau, (**H**) p-tau (S202), and (**I**) p-tau (S202)/t-tau. ROC analysis revealed moderate diagnostic accuracy of elevated serum t-tau. AUC, area under the curve.

We first measured serum concentrations of t-tau and p-tau in all subjects by enzyme-linked immunosorbent assays (ELISAs) to examine the potential of the proteins as non-invasive biomarkers for AD (Figure 1). Indeed, serum t-tau was significantly higher in the Mild-AD group compared to AMCs (351.9 ± 50.04 pg/mL vs. 245.6 ± 33.76 pg/mL; *p* < 0.05, Figure 1A), while concentration in the MCI group did not differ from AMCs (263.0 ± 37.12 pg/mL). Serum p-tau (pSer202: S202) was also slightly higher in the MCI and Mild-AD groups compared to the AMCs, but the differences did not reach significance (AMC, 98.60 ± 16.23; MCI, 127.0 ± 20.07; Mild-AD, 120.1 ± 17.84, *p =* 0.38, Figure 1B). The serum p-tau (S202)/t-tau protein ratio also did not differ among groups (AMC, 0.36 ± 0.03; MCI, 0.45 ± 0.03; Mild-AD, 0.35 ± 0.04, *p =* 0.799, Figure 1C). These results suggest that serum t-tau may distinguish Mild-AD but not MCI from age-matched health subjects.

Next, we evaluated the correlations between serum t-tau levels and neurocognitive test scores because only serum t-tau was significantly higher in the AD groups according to ELISA results. Serum t-tau concentration exhibited a weak negative correlation with MMSE score (r = −0.19, *p* = 0.11, Figure 1D) and positive correlation with GDS score (r = 0.22, *p* = 0.06, Figure S1A) but no correlation with CDR-SOB score (r = 0.13, *p* = 0.27, Figure S1D). There were also no significant correlations between serum p-tau (S202) levels or p-tau (S202)/t-tau ratio and neurocognitive test scores (Figure 1E,F and Figure S1B,C,E,F). Moreover, there was no correlation between serum t-tau or p-tau (S202) and age (t-tau, r = 0.05, *p* = 0.66, Figure S1G; p-tau, r = 0.02, *p* = 0.88, Figure S1H) despite the strong influence of age on AD risk.

We also performed Receiver operating characteristic (ROC) analysis to evaluate the diagnostic utility of serum t-tau and p-tau. Serum t-tau elevation above 234.4 pg/mL distinguished Mild-AD from AMC group subjects with 75% sensitivity and 61.54% specificity (area under the curve (AUC) = 0.675, *p* = 0.044, Figure 1G), while serum p-tau (S202) above 58.34 pg/mL distinguished Mild-AD from AMC subjects with 78.95% sensitivity and but only 40% specificity (AUC = 0.5958, *p* = 0.281, Figure 1H). Serum p-tau (S202)/t-tau ratio also was not a reliable marker, distinguishing Mild-AD from AMC group subjects with 42.11% sensitivity and 76% specificity using a cut-off of 0.245 (AUC = 0.525, *p* = 0.78, Figure 1I). Therefore, a rise in t-tau or p-tau distinguished mild-AD from healthy age-matched controls with only moderate accuracy. However, we speculated that tau proteins in brain-derived exosomes more accurately reflect current disease status that total serum proteins.

### 2.2. Characteristics of Neuronal Cell-Derived Exosomes (NEX)

To determine whether serum tau proteins originate from neuronal cells and enter the circulation via neuronal-derived exosomes (NEX), we isolated exosomes from serum using the ExoQuick EX precipitation solution according to Perez-Gonzalez [27] with minor modifications and then enriched (NEX) by immunochemical methods (Figure S2A). To ensure the identity and quality of EX and NEX, we characterized the microvesicles by NanoSight, Western blotting, and transmission electron microscopy (TEM). NanoSight results showed that particles in the ExoQuick precipitates (total serum EX) ranged in diameter from 83 to 159 nm, consistent with expected EX size (Figure S2C). Further, EX identity and NEX enrichment were confirmed by Western blot detection of the EX-specific protein marker CD63 and the neuronal marker NCAM-L1 (Figure S2D). Both CD63 and NCAM-L1 were expressed in the total EX fraction (initial ExoQuick precipitate) and the NEX fraction (after immuno-enrichment) from the serum of AD patients and CTL subjects (Figure S2D). Expression of NCAM-LI was higher in the NEX fraction, consistent with a neuronal origin, whereas CD63 expression was lower than in the total EX faction (EX + NEX), consistent with enrichment (Figure S2D). In addition, consistent with a brain origin of NEX isolated from serum, TEM analysis of both exosomes isolated from serum and brain tissue samples revealed similar circular structures within the same diameter range of 50 to 150 nm (Figure S2E). These combined morphometric and immunolabeling results confirmed the successful isolation of exosomes from brain and serum as well as the neural origin of the serum NEX fraction.

### 2.3. NEX t-tau and p-tau Protein Levels in Controls, Mild Cognitive Impairments, and Mild-AD Patients

**Figure 2:**
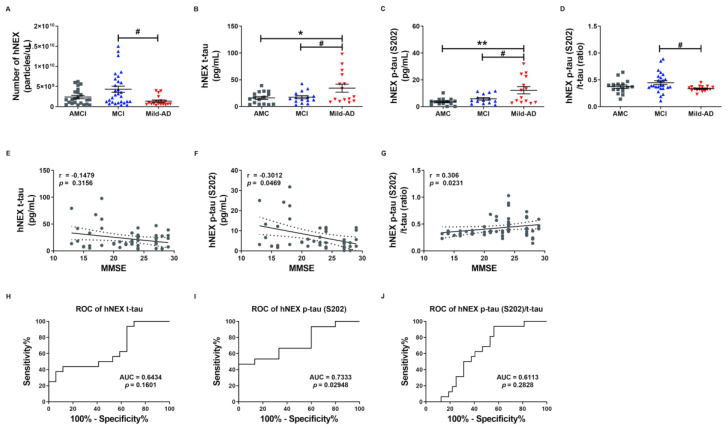
Serum total tau and phosphorylated tau in neuronal cell-derived exosomes are elevated according to the severity of Alzheimer’s disease. (**A**) The number of human neuronal cell-derived exosomes (hNEX) was quantified by ELISA for the exosome marker CD63. The number of hNEX was lower in the Mild-AD group than the MCI group. (**B**) Total tau (t-tau), (**C**) p-tau (S202), and (**D**) p-tau (S202)/t-tau ratio in human neuronal cell-derived exosomes (hNEX) were quantified using ELISA. hNEX t-tau and p-tau (S202) were higher in the Mild-AD group than the AMC and MCI groups. All data were shown as means ± SEM. * *p* < 0.05 and ** *p* < 0.01 compared to the AMC group and ^#^ *p* < 0.05 compared to the MCI group by one-way ANOVA and Holm-Sidak’s or Dunn’s multiple comparison test. Correlations of hNEX (**E**) t-tau, (**F**) p-tau (S202), and (**G**) p-tau (S202)/t-tau with MMSE scores were assessed using the nonparametric Spearman’s rank correlation test. Graphs show regression lines with 95% confidence intervals. hNEX p-tau (S202) and p-tau (S202)/t-tau were significantly correlated with MMSE scores. ROC analyses of hNEX (**H**) t-tau, (**I**) p-tau (S202), and (**J**) p-tau (S202)/t-tau indicating moderate diagnostic accuracy of elevated hNEX p-tau (S202). AUC, area under the curve.

To investigate whether serum NEX tau proteins more accurately reflect the severity of AD than total serum tau proteins, we measured t-tau and p-tau levels in suspensions of human neural exosomes (hNEX) from the AMC (*n* = 23), MCI (*n* = 29), and Mild-AD (*n* = 18) groups by ELISA. The variation in EX yield was controlled by normalizing hNEX number to EX marker CD63 immunoreactivity. The number of hNEX in the Mild-AD group was significantly lower than in the MCI group (1.38 × 10^9^ ± 2.87 × 10^8^ vs. 4.32 × 10^9^ ± 7.67 × 10^8^; *p* < 0.05, Figure 2A) but did not differ from the AMC group (2.39 × 10^9^ ± 4.04 × 10^8^).

The expression profile of these EX proteins was distinct in AD patients compared to the AMC group. The hNEX t-tau level was significantly higher in the Mild-AD group than the AMC and MCI groups (AMC, 16.16 ± 2.81; MCI, 17.45 ± 2.84; Mild-AD, 34.28 ± 7.58, both *p* < 0.05, Figure 2B). Similarly, the hNEX p-tau (S202) level was significantly higher in the Mild-AD group than the MCI and AMC groups (AMC, 3.63 ± 0.70; MCI, 5.85 ± 0.96; Mild-AD, 12.24 ± 2.69, *p* < 0.01 vs. AMC, *p* < 0.05 vs. MCI, Figure 2C). In addition, the hNEX p-tau (S202)/t-tau ratio was significantly higher in the MCI compared to the AMC group (AMC, 0.37 ± 0.03; MCI, 0.45 ± 0.036; Mild-AD, 0.34 ± 0.01, *p* < 0.05 vs. MCI, Figure 2D).

Next, we evaluated the correlations between hNEX tau levels and neurocognitive test scores and found that hNEX p-tau (S202) levels were negatively correlated with MMSE scores (r = −0.30, *p* = 0.05, Figure 2F) and demonstrated weak positive correlations with GDS scores (r = 0.27, *p* = 0.07, Figure S3B) and CDR-SOB scores (r = 0.27, *p* = 0.07, Figure S3E). In contrast, there were no correlations between hNEX t-tau levels and neurocognitive test scores (Figure 1E and Figure S3A,D). However, the hNEX p-tau (S202)/t-tau ratio was positively correlated with MMSE scores (r = 0.30, *p* = 0.02, Figure 2G) and demonstrated a weak negative correlation with CDR-SOB scores (r = −0.26, *p* = 0.06, Figure S3F), but no correlation with GDS scores (r = −0.19, *p* = 0.19, Figure S3C). No correlation between hNEX t-tau level or p-tau and age was detected (t-tau, r = 0.08903, *p* = 0.5025, Figure S3G; p-tau, r = −0.02038, *p* = 0.8720, Figure S3H).

We then performed ROC analysis to evaluate the diagnostic accuracy of hNEX t-tau and found that a level higher than 30.94 pg/mL distinguished Mild-AD from AMC group subjects with a sensitivity of 43.75% and specificity of 88.24% (AUC = 0.6434, *p* = 0.16, Figure 2H). Notable, however, hNEX p-tau (S202) above 11.85 pg/mL distinguished Mild-AD from AMC subjects with 46.67% sensitivity but 100% specificity (AUC = 0.73, *p* = 0.03, Figure 2I), and a hNEX p-tau (S202)/t-tau of 0.39 distinguished Mild-AD from AMC subjects with 93.75% sensitivity and 43.75% specificity (AUC = 0.6113, *p* = 0.28, Figure 2J). These findings suggest that hNEX p-tau (S202)/t-tau ratio may serve as reliable biomarkers for Mild-AD.

We also evaluated p-tau (pThr181: T181) and Aβ levels in hNEX and serum as these proteins are strongly implicated in AD pathogenesis. There were no significant differences in p-tau (T181) among groups (serum: AMC, 9.59 ± 0.40; MCI, 9.68 ± 0.39; Mild-AD, 9.86 ± 0.69, Figure S4A; hNEX: AMC, 14.11 ± 2.68; MCI, 14.06 ± 3.04; Mild-AD, 22.05 ± 4.04, Figure S4D). Alternatively, serum Aβ was significantly lower in the Mild-AD group compared to the MCI group, while hNEX Aβ was slightly greater in the Mild-AD group compared to both MCI and AMC groups (serum: AMC, 23.10 ± 1.29; MCI, 27.14 ± 0.88; Mild-AD, 19.22 ± 2.24, *p* < 0.001 vs. MCI, Figure S4B; hNEX: AMC, 4.25 ± 0.64; MCI, 4.08 ± 1.07; Mild-AD, 6.18 ± 0.94, Figure S4E). We also evaluated the serum and hNEX p-tau (T181)/Aβ_1-42_ ratio level, but found no significant differences among groups (serum: AMC, 0.44 ± 0.04; MCI, 0.37 ± 0.02; Mild-AD, 0.52 ± 0.06, Figure S4C; hNEX: AMC, 4.70 ± 0.64; MCI, 4.37 ± 0.40; Mild-AD, 3.62 ± 0.43, Figure S4F). Next, we evaluated the correlation between serum Aβ levels and neurocognitive test scores but found no significant correlations with MMSE scores (r = 0.12, *p* = 0.30, Figure S4G), GDS scores (r = −0.13, *p* = 0.27, Figure S4H), or CDR-SOB scores (r = −0.04, *p* = 0.72, Figure S4I).

Females are known to have a higher incidence of AD than males, but there were no significant sex differences in serum t-tau (Male, 251.7 ± 32.40; Female, 301.1 ± 31.97, Figure S5A), p-tau (S202) (Male, 117.8 ± 16.43; Female, 113.3 ± 14.08, Figure S5B), and p-tau (T181) (Male, 9.65 ± 0.48; Female, 9.73 ± 0.33, Figure S5C). Similarly, there were no significance sex differences in hNEX t-tau (Male, 22.84 ± 2.79; Female, 19.39 ± 1.99, Figure S5E) and p-tau (T181) level (Male, 22.52 ± 2.76; Female, 17.67 ± 1.84, Figure S5G), but hNEX p-tau (S202) was significantly lower in females (Male, 14.85 ± 2.07; Female, 9.84 ± 1.42, *p* < 0.05, Figure S5F). We also evaluated Aβ levels in serum and hNEX of males and females but found no significant sex differences (serum: Male, 25.15 ± 1.14; Female, 23.14 ± 1.20, Figure S5D; hNEX: Male, 5.11 ± 0.85; Female, 4.03 ± 0.52, Figure S5H).

### 2.4. Phosphorylated tau Protein Levels in Serum and hNEX Predict Cognitive Deterioration

**Figure 3:**
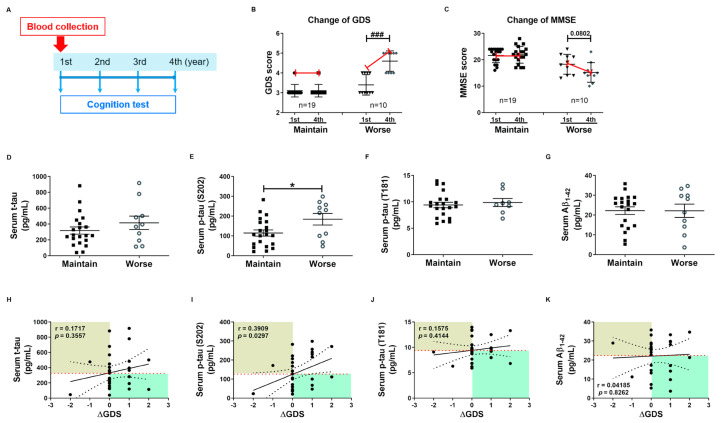
High baseline p-tau in serum predicts long-term cognitive deterioration. (**A**) Timeline of the follow-up study. Blood was collected once during the first year, and cognitive function tests were performed annually for 4 years. Changes in (**B**) GDS score (ΔGDS) and (**C**) ΔMMSE score are indicative of cognitive deterioration. All data were shown as means ± SEM. ^###^ *p* < 0.001 compared to the first-year score in the cognitive deterioration group using the Mann–Whitney test. Comparisons of baseline serum (**D**) t-tau, (**E**) p-tau (S202), (**F**) p-tau (T181), and (**G**) Aβ_1–42_ between slow progression and cognitive deterioration groups. Serum p-tau (S202) levels were higher in the cognitive deterioration group than the slow progression group. All data were shown as means ± SEM. * *p* < 0.05 compared to the slow progression group using the Mann–Whitney test. Correlations of serum (**H**) t-tau, (**I**) p-tau (S202), (**J**) p-tau (T181), and (**K**) Aβ_1–42_ with ΔGDS were assessed using the nonparametric Spearman’s rank correlation test. Serum p-tau (S202) levels were significantly correlated with ΔGDS scores.

**Figure 4:**
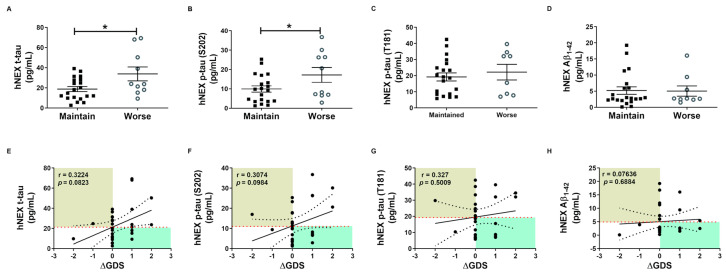
Total tau and phosphorylated tau protein levels in neuronal cell-derived exosomes predict cognitive deterioration. Comparisons of baseline hNEX (**A**) t-tau, (**B**) p-tau (S202), (**C**) p-tau (T181), and (**D**) Aβ_1-42_ between slow progression and cognitive deterioration groups. Baseline hNEX t-tau and p-tau (S202) were higher in the cognitive deterioration group than the slow progression group. All data were shown as means ± SEM. * *p* < 0.05 compared to the slow progression group by Mann–Whitney test. Correlations of hNEX (**E**) t-tau, (**F**) p-tau (S202), (**G**) p-tau (T181), and (**H**) Aβ_1-42_ with ΔGDS were assessed using the nonparametric Spearman’s rank correlation test.

These tau protein measures and correlations with AD severity are from patients at different stages of AD, and thus associations with diagnostic significance may be overlooked. Therefore, we examined these associations prospectively during patient follow-up. Blood samples were collected only in the first year, and cognitive function tests were performed annually for 4 years. Changes in GDS and MMSE scores were used for the evaluation of cognitive deterioration. Based on these results, patients were divided into a slow progression group showing no significant increase in mean GDS score or a significant decrease in mean MMSE score, and a cognitive deterioration group demonstrating significantly higher mean GDS scores (1st, 3.40 ± 0.16; 4th, 4.60 ± 0.16, *p* < 0.01, Figure 3B) and numerically lower mean MMSE score (1st, 18.30 ± 1.19; 4th, 15.20 ± 1.17, *p* = 0.08, Figure 3C). Serum p-tau (S202) levels were higher in the cognitive deterioration group than the slow progression group (184.2 ± 29.29 vs. 114.6 ± 15.64 pg/mL, *p* < 0.05, Figure 3E), and there was a significant positive correlation between serum p-tau (S202) and the change in GDS score (ΔGDS) (r = 0.3909, *p* = 0.0297, Figure 3I). Alternatively, there were no group differences in t-tau, p-tau (T181), and Aβ_1-42_ or correlations of these factors with ΔGDS (Figure 3H–K). Baseline hNEX t-tau level was also greater in the cognitive deterioration group than the slow progression group (33.83 ± 6.90 vs. 18.78 ± 2.46 pg/mL, *p* < 0.05, Figure 4A). Similarly, hNEX p-tau (S202) level was higher in the cognitive deterioration group compared to the slow progression group (17.20 ± 3.84 vs. 9.94 ± 1.63; *p* < 0.05, Figure 4B). Thus, elevated hNEX t-tau, hNEX p-tau (S202), and serum p-tau (S202) are predictive of cognitive deterioration. In contrast, hNEX t-tau, p-tau (S202), p-tau (T181), and Aβ_1-42_ were not correlated with ΔGDS (Figure 4E–H). In addition, t-tau, p-tau (T181), and Aβ_1-42_ in human serum and neuronal cell-derived exosomes were not correlated with ΔMMSE score (Figure S6), but serum p-tau (S202) was negatively correlated with ΔMMSE score (r = −0.35, *p* = 0.05, Figure S6B).

The expression of tau proteins in hNEX was confirmed by Western blot analysis (Figure S7A). hNEX t-tau expression was significantly higher in the Mild-AD group than the AMC group (AMC, 1.000 ± 0.1328; MCI, 0.98 ± 0.16; Mild-AD, 1.43 ± 0.08, *p* < 0.05 vs. AMC, Figure S7B). The Mild-AD group also exhibited significantly higher hNEX hyper-phosphorylated tau (p-tau (S202, T205)) (AMC, 1.00 ± 0.09; MCI, 0.81 ± 0.14; Mild-AD, 1.91 ± 0.16, *p* < 0.05 vs. AMC, *p* < 0.01 vs. MCI, Figure S7C) and p-tau (T181) (AMC, 1.00 ± 0.17; MCI, 1.22 ± 0.23; Mild-AD, 2.66 ± 0.33, *p* < 0.01 vs. AMC, *p* < 0.05 vs. MCI, Figure S7D), while hNEX p-tau (T231) did not differ between groups (AMC, 1.00 ± 0.10; MCI, 1.30 ± 0.16; Mild-AD, 1.12 ± 0.16, *p* < 0.01, Figure S7E). The Mild-AD group also demonstrated a significantly higher hNEX p-tau (S202, T205)/t-tau ratio than the MCI group (AMC, 1.00 ± 0.06; MCI, 0.84 ± 0.06; Mild-AD, 1.30 ± 0.15, *p* < 0.01 vs. MCI, Figure S7G) and a higher hNEX p-tau (T181)/t-tau ratio than the AMC group (AMC, 1.00 ± 0.11; MCI, 1.47 ± 0.16; Mild-AD, 2.07 ± 0.32, *p* < 0.01 vs. AMC, Figure S7H).

### 2.5. Phosphorylated Tau Protein Expression in Brain Tissue Exosomes of JNPL3 Mice Increased with The Progression of Pathology

**Figure 5:**
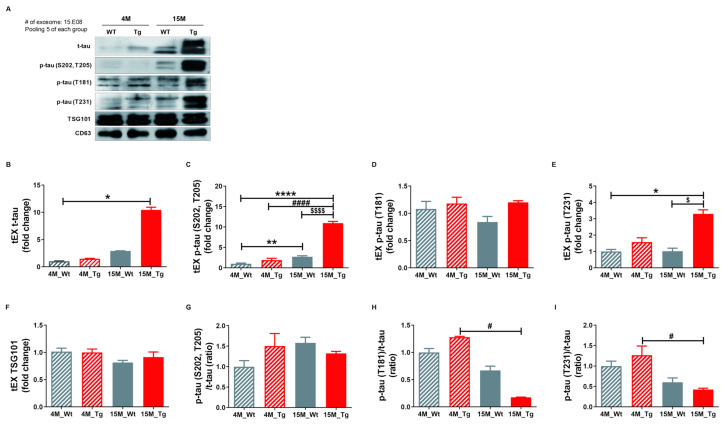
Total tau and phosphorylated tau expression levels in brain tissue exosomes of JNPL3 mice increase with the progression of pathology. Total tau and phosphorylated tau protein expression in brain tissue exosomes (tEX) were validated by Western blot. (**A**) Representative Western blot. Relative expression levels of (**B**) t-tau, (**C**) hyperphosphorylated tau (p-tau (S202, T205)), (**D**) p-tau (T181), (**E**) p-tau (T231), and (**F**) TSG101 as well as the (**G**) p-tau (S202, T205)/t-tau ratio, (**H**) p-tau (T181)/t-tau ratio, and (**I**) p-tau (T231)/t-tau ratio in tEX. tEX t-tau was higher in 15-month-old JNPL3 mice (15M-Tg) than 4-month-old wild type mice (4M-WT). tEX p-tau (S202, T205) was higher in 15M-Tg mice than 4M-WT, 4M-Tg, and 15M-WT mice. tEX p-tau (T231) was higher in 15M-Tg mice than 4M-WT and 15M-WT mice. tEX p-tau (T181)/t-tau and p-tau (T231)/t-tau were lower in 15M-Tg mice than 4M-Tg mice. All data were shown as means ± SEM. * *p* < 0.05, ** *p* < 0.01, and **** *p* < 0.0001 compared to 4M-WT, ^#^ *p* < 0.05 and ^####^ *p* < 0.0001 compared to 4M-Tg, ^$^ *p* < 0.05 and ^$$$$^ *p* < 0.0001 compared to 15M-WT mice by one-way ANOVA and Dunn’s multiple comparison test. 4M-WT (*n* = 10), 4M-Tg (*n* = 8), 15M-WT (*n* = 8), and 15M-Tg (*n* = 7).

To confirm our hypothesis that NEX isolated from blood originate as brain tissue exosomes that cross the blood-brain barrier (BBB) and thus reflect the current disease state, we examined the protein expression profiles of these vesicles in the JNPL3 transgenic (Tg) mouse model of AD (Tg-4M and Tg-15M) and wild type littermates (WT-4M and WT-15M). Mutant mice were distinguished from WTs by genotyping (Figure S8A) and immunohistochemistry using the AT8 antibody (Figure S8B). Relative expression levels of t-tau, p-tau (S202, T205), p-tau (T181), p-tau (T231), and TSG101 as well as the p-tau (S202, T205)/t-tau, p-tau (T181)/t-tau, and p-tau (T231)/t-tau ratios in tEX were examined by Western blotting (Figure 5A–I). Expression of tEX t-tau was higher in 15M-WT mice than 4M-WT mice (2.92 ± 0.01 vs. 1.00 ± 0.11; ns, Figure 5B) and 15M-Tg mice compared to 4M-Tg (10.44 ± 0.50 vs. 1.48 ± 0.08, ns, Figure 5B), and markedly higher in 15M-Tg mice compared to both 4M-WT mice (*p* < 0.05). The dramatic accumulation of t-tau in EX during age was consistent with the pathological progression of AD. Similarly, tEX p-tau (S202, T205) was significantly higher in 15M-Tg mice compared to 4M-WT, 4M-Tg, and 15M-WT mice (4M-WT, 1.01 ± 0.16; 4M-Tg, 1.90 ± 0.44; 15M-WT, 2.72 ± 0.23; 15M-Tg, 10.96 ± 0.42, *p* < 0.0001 and *p* < 0.01 vs. 4M-WT, *p* < 0.0001 vs. 4M-Tg, *p* < 0.0001 vs. 15M-WT, Figure 5C), while tEX p-tau (T231) was higher in 15M-Tg mice compared to 4M-WT and 15M-WT mice (4M-WT, 1.00 ± 0.13; 4M-Tg, 1.58 ± 0.27; 15M-WT, 1.03 ± 0.18; 15M-Tg, 3.30 ± 0.25, *p* < 0.05 vs. 4M-WT, *p* < 0.05 vs. 15M-WT, Figure 5E). Alternatively, 15M-Tg mice exhibited the lowest tEX p-tau (T181)/t-tau ratio (4M-WT, 1.00 ± 0.07; 4M-Tg, 1.28 ± 0.02; 15M-WT, 0.67 ± 0.08; 15M-Tg, 0.18 ± 0.00, *p* < 0.05 vs. 4M-Tg, Figure 5H) and a lower p-tau (T231)/t-tau ratio compared to 4M-Tg mice (4M-WT, 1.00 ± 0.12; 4M-Tg, 1.27 ± 0.23; 15M-WT, 0.60 ± 0.10; 15M-Tg, 0.42 ± 0.032, *p* < 0.05 vs. 4M-Tg, Figure 5I).

### 2.6. Total Tau and Phosphorylated Tau Protein Levels in Blood NEX from JNPL3 Mice Increased with The Progression of Pathology

**Figure 6:**
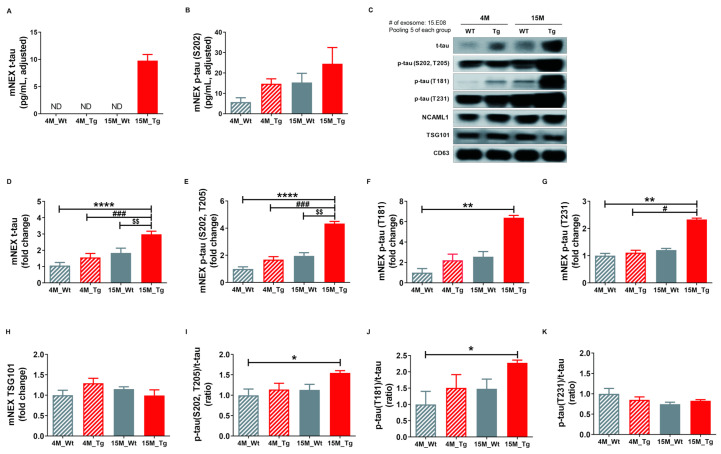
Total tau and phosphorylated tau protein levels in NEX from the blood of JNPL3 mice increase with the progression of pathology. (**A**) Total tau (t-tau) and (**B**) p-tau (S202) in mouse neuronal cell-derived exosomes (mNEX) were quantified using ELISA. 4M-WT (*n* = 5), 4M-Tg (*n* = 10), 15M-WT (*n* = 11), and 15M-Tg (*n* = 17). (**C**–**K**) mNEX t-tau and p-tau (S202) were higher in 15M-Tg mice. mNEX t-tau and p-tau expression were validated by Western blot. **C**) Representative Western blot. Relative expression levels of (**D**) t-tau, (**E**) hyperphosphorylated tau (p-tau (S202, T205)), (**F**) p-tau (T181), (**G**) p-tau (T231), and (**H**) TSG101 as well as (**I**) p-tau (S202, T205)/t-tau ratio, (**J**) p-tau (T181)/t-tau ratio, and (**K**) p-tau (T231)/t-tau ratio in mouse NEX (mNEX). mNEX t-tau and p-tau (S202, T205) were higher in 15M-Tg mice compared to 4M-WT, 15M-WT, and 15M-Tg mice. mNEX p-tau (T181), p-tau (S202, T205)/t-tau ratio, and p-tau (T181)/t-tau ratio were higher in 15M-Tg mice compared to 4M-WT mice. mNEX p-tau (T231) was higher in 15M-Tg mice compared to 4M-WT and 4M-Tg mice. All data were shown as means ± SEM (*n* = 14 per group). * *p* < 0.05, ** *p* < 0.01, and **** *p* < 0.0001 compared to 4M-WT, ^#^ *p* < 0.05 and ^###^ *p* < 0.001 compared to 4M-Tg, and ^$$^ *p* < 0.01 compared to 15M-WT mice by one-way ANOVA and Bonferroni’s multiple comparison test.

Finally, we quantified total tau (t-tau) and p-tau (S202) in mouse neuronal cell-derived exosomes (mNEX) from 4- or 15-month-old WT and Tg mice using ELISA. mNEX t-tau and p-tau (S202) were significantly higher in 15M-Tg mice than all other groups (Figure 6A,B) and these differences were validated by Western blot (Figure 6C). Relative expression levels of t-tau, p-tau (S202, T205), p-tau (T181), p-tau (T231), and TSG101, as well as p-tau (S202, T205)/t-tau, J) p-tau (T181)/t-tau, and K) p-tau (T231)/t-tau ratios in mNEX were also examined (Figure 6D–K). The 15M-Tg group demonstrated highest mNEX expression levels of t-tau (4M-WT, 1.06 ± 0.19; 4M-Tg, 1.57 ± 0.24; 15M-WT, 1.84 ± 0.29; 15M-Tg, 2.98 ± 0.19, *p* < 0.0001 vs. 4M-WT, *p* < 0.001 vs. 4M-Tg, *p* < 0.01 vs. 15M-WT, Figure 6D) and p-tau (S202, T205) (4M-WT, 1.00 ± 0.15; 4M-Tg, 1.68 ± 0.22; 15M-WT, 1.96 ± 0.24; 15M-Tg, 4.34 ± 0.15, *p* < 0.0001 vs. 4M-WT, *p* < 0.001 vs. 4M-Tg, *p* < 0.01 vs. 15M-WT, Figure 6E). The 15M-Tg group also exhibited greater expression of mNEX p-tau (T181) compared to 4M-WT mice (4M-WT, 1.00 ± 0.40; 4M-Tg, 2.22 ± 0.60; 15M-WT, 2.56 ± 0.51; 15M-Tg, 6.39 ± 0.23, *p* < 0.01 vs. 4M-WT, Figure 6F) and greater mNEX p-tau (T231) expression compared to 4M-WT and 4M-Tg mice (4M-WT, 1.00 ± 0.08; 4M-Tg, 1.11 ± 0.09; 15M-WT, 1.21 ± 0.06; 15M-Tg, 2.33 ± 0.05, *p* < 0.01 vs. 4M-WT, *p* < 0.05 vs. 4M-Tg, Figure 6G). Ratios of p-tau (S202, T205)/t-tau (4M-WT, 1.00 ± 0.15; 4M-Tg, 1.14 ± 0.15; 15M-WT, 1.13 ± 0.14; 15M-Tg, 1.55 ± 0.05, *p* < 0.05 vs. 4M-WT, Figure 6I) and p-tau (T181)/t-tau were also higher in the 15M-Tg group compared to the 4M-WT group (4M-WT, 1.00 ± 0.40; 4M-Tg, 1.51 ± 0.41; 15M-WT, 1.48 ± 0.29; 15M-Tg, 2.28 ± 0.08, *p* < 0.05 vs. 4M-WT, Figure 6J).

**Figure 7:**
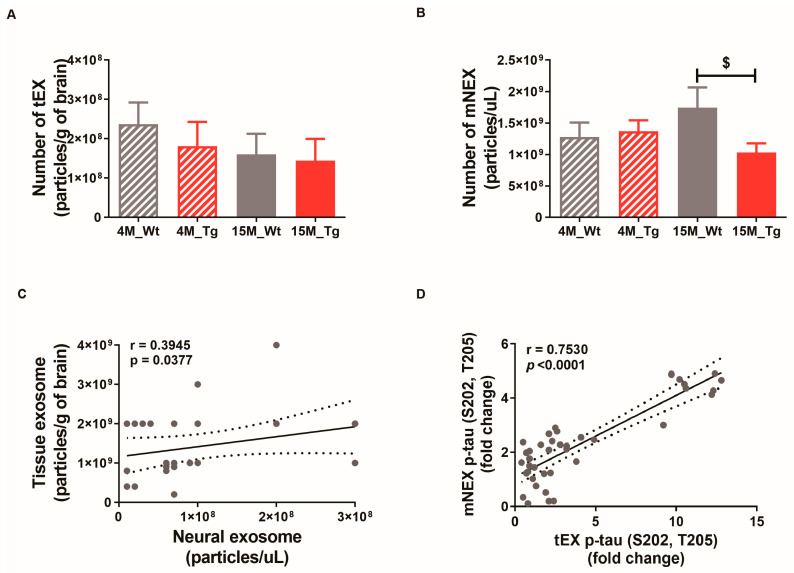
Correlations of neural exosome and brain tissue exosome numbers and protein contents in JNPL3 mice. Number of (**A**) tEX and (**B**) mNEX were quantified by ELISA for CD63. The number of mNEX was lower in 15M-Tg mice than 15M-WT mice. All data were shown as means ± SEM. 4M-WT (*n* = 10), 4M-Tg (*n* = 8), 15M-WT (*n* = 8), and 15M-Tg (*n* = 7) in tEX analysis. 4M-WT (*n* = 8), 4M-Tg (*n* = 10), 15M-WT (*n* = 11), and 15M-Tg (*n* = 17) in mNEX analysis. ^$^ *p* < 0.05 compared to the 15M-WT group by one-way ANOVA and Dunn’s Multiple comparison test. (**C**) The correlation between tEX and mNEX was assessed using the nonparametric Spearman’s rank correlation test. Graph shows regression line with 95% confidence interval. (**C**,**D**) Both the number of particles and hyperphosphorylated tau (p-tau (S202, T205)) content was correlated between tEX and mNEX.

The numbers of tEX, mNEX, and hNEX were quantified by ELISA for total CD63 expression (Figure 7A,B). The number of mNEX was significantly lower in 15M-Tg mice compared to 15M-WT mice (4M-WT, 1.28 × 10^9^ ± 2.33 × 10^8^; 4M-Tg, 1.37 × 10^9^ ± 1.73 × 10^8^; 15M-WT, 1.75 × 10^9^ ± 3.20 × 10^8^; 15M-Tg, 1.03 × 10^9^ ± 1.46 × 10^8^, *p* < 0.05 vs. 15M-WT, Figure 7B). These results suggest that the number of hNEX decreases during the progression from MCI to mild-AD (Figure 2A). As expected, both tEX and mNEX were also significantly correlated with total particle number (r = 0.39, *p* < 0.05, Figure 7C) and p-tau (S202, T205) expression (r = 0.75, *p* < 0.0001, Figure 7D).

## 3. Discussion

In this study, we found significantly elevated total tau protein in the serum of Mild-AD patients compared to MCI group and AMC group, but this elevation was only of modest efficacy for identifying AD patients according to ROC analysis. The tau protein has more than 25 phosphorylation sites [7], and changes in pSer202 (p-tau (S202)) and pThr181 (p-tau (T181)) are implicated in AD [26]. However, no significant changes in serum p-tau (S202) and p-tau (T181) were observed among AD groups compared to healthy controls. We did find significant correlations of serum t-tau levels with MMSE and GDS scores and no age- or gender-dependent changes, indicating specific dependence on disease progression. However, these correlations were not strong, possibly due to the absence of severely symptomatic patients in our sample. There was also no correlation between t-tau and CDR-SOB scores. The pathophysiological process of AD is thought to begin several years before clinical symptoms become apparent [28]; therefore, CDR-SOB scores are believed to follow neurodegenerative changes. In our study, the CDR-SOB score was measured once when patients visited the hospital voluntarily, but a single measure was insufficient to identify mild-AD, which may explain why there were no correlations with other disease markers. In addition to the low-to-moderate accuracy, sensitivity, and specificity of serum t-tau for AD diagnosis, we found no changes in serum Aβ or correlations with neurocognitive test scores. In summary, there were significant differences in serum tau between AMC and Mild-AD, although there was no clear difference between MCI and Mild-AD groups. However, serum Aβ levels decreased with disease progression from MCI to mild-AD. Until now, there have been numerous studies searching for useful blood-based AD-biomarkers, including plasma Aβ or tau. A recent study using ultrasensitive methods showed that plasma Aβ was associated with cognitive status and CSF biomarkers and plasma Aβ_42_ and Aβ_40_ were lower in AD than in amnestic MCI than in non-amnestic MCI [29], whereas others reported the opposite [30,31,32,33,34]. These equivocal findings yield conflicting results concerning the predictive value of AD diagnosis and cognitive decline in the AD group. Recent reports regarding tau levels revealed that plasma tau reflected brain tau levels [17], and plasma tau levels were specifically elevated in AD patients [19]. At the same time, other studies concluded that t-tau were not suitable as AD biomarkers because of the large overlap of plasma t-tau levels between normal aging and AD [19,20]. A more recent study quantifying plasma p-tau (T181) showed that the plasma p-tau (T181) in the AD group was significantly higher than that in the age-matched control group, but showed a too low cut-off value (0.0921 pg/mL) of plasma p-tau181 [20] This result with plasma p-tau (T181) differs from our ELISA results that serum p-tau (T181) did not differ between AMC, MCI, and Mild-AD. In addition, the scale of the measured value fo p-tau was different. Perhaps this discrepancy is due to differences in the experimental scheme, including blood samples and measurement methods. However, our WB results for p-tau (T181) levels show that p-tau (T181) protein in Mild-AD was significantly increased compared to both the MCI and AMC groups. P-tau (T181)/t-tau ratio of the Mild-AD group also significantly higher than that of the AMC group. Since the results seem to differ depending on the experiment method, the potential for AD biomarkers of plasma t-tau and p-tau requires further examination for diagnostic reliability, including correlation with clinical features such as cognitive dysfunction.

In contrast to total serum tau proteins, we found that serum exosome tau was more strongly predictive of disease status according to ROC, presumably as these vesicle-associated proteins better reflect the pathological status of the brain. Tau proteins are abundant in the brain, especially in distal axons [7]. We hypothesized that pathological proteins such as t-tau and p-tau can be delivered to the circulation by exosomes, which can act as biological barrier-permeable carriers. Therefore, we isolated human neural exosomes (hNEX) in serum samples from the AMC, MCI, and Mild-AD group subjects and measured expression levels of tau protein by ELISA and Western blot. Although the level of tau protein in NEX was about one-tenth that in serum, group differences were more pronounced. For instance, while serum p-tau did not differ among groups (Figure 1), serum hNEX p-tau was significantly higher in the Mild-AD group than the MCI and AMC groups, while the hNEX p-tau/t-tau ratio was higher in the Mild-AD group than the AMC group, respectively. Further, both NEX t-tau and p-tau (S202) protein levels distinguished MCI from mild-AD with high specificity or sensitivity according to ROC analysis. NEX p-tau (S202) was also significantly correlated with MMSE scores, further suggesting a strong association with disease progression.

As the variability of cross-sectional data can overlook clinically useful associations, enrolled subjects were also followed for 4 years with annual cognitive function testing following a single blood test. Both serum and NEX p-tau (S202) during the first year were correlated with the changes in GSD and MMSE scores after 4 years, indicating that high baseline serum or NEX p-tau protein is predictive of faster disease progression and cognitive decline. Alternatively, p-tau (T181) and Aβ levels did not correlate with these changes in GSD and MMSE scores during follow-up.

Both t-tau and p-tau proteins were significantly elevated in the Mild-AD group, as confirmed by Western blotting. In particular, PHF (pSer202 + pThr205) and pThr181 were significantly higher in the Mild-AD group. These results clearly show that at least some pathogenic proteins in the brain tissue can enter the circulation via exosomes, thus reflecting the current state of brain pathology. It has been reported that tau protein is difficult to measure in blood because of its short half-life [35,36]. However, pathogenic proteins in blood may be protected from enzymatic damage if contained within vesicles such as exosomes. Although NEX tau proteins can distinguish between MCI and mild-AD more accurately than the corresponding free serum proteins, there are several drawbacks. First, the isolation process is complex. Further, individual proteins only partially reflect the complex pathology of AD. A combination of NEX proteins may provide a more accurate and reliable diagnosis, disease staging, and prognosis.

Since NEX isolated from blood is derived from brain tissue exosomes, we hypothesized that the changes in NEX proteins would reflect pathogenic changes in the brain. There was a significant decrease in the number of hNEX in the Mild-AD group compared to the MCI group as well as in aged AD model mice compared to younger WT and Tg mice, suggesting reduced tau protein clearance in the brain with disease progression and that exosomal egress serves a protective function. In addition to pathogenic effects, tau proteins remaining in the brain may facilitate further accumulation. This possibility was confirmed using JNPL3 Tg mice, in which p-tau accumulation was greater in the brains of 15-month-old than 4-month-old animals. In accord with human blood NEX, the number of mouse neural exosomes (mNEX) was significantly lower in 15-month-old Tg JNPL3 mice compared to age-matched WT mice. We also found that the number of neural exosomes was significantly correlated with the number of brain tissue exosomes. Expression levels of t-tau and p-tau proteins in blood NEX as well as brain tissue exosomes increased with pathological progression in JNPL3 mice. Moreover, the expression of NEX p-tau protein was significantly correlated with p-tau protein in brain exosomes. Collectively, these findings indicate that tau proteins in blood exosomes reflect the level of tau proteins in the brain, and thus may be useful markers for monitoring the progression of AD.

In this study, we demonstrate that total tau and phospho-tau (S202) associated with brain-derived serum exosomes can distinguish mild AD from MCI and healthy controls with greater accuracy than free serum proteins. Further, we show that elevated levels of these serum exosome-associated proteins at baseline can predict long-term cognitive decline. Finally, we provide compelling evidence from AD model mice that AD-related proteins in serum exosomes are indeed derived from brain exosomes.

## 4. Materials and Methods

### 4.1. Patients, Controls, and Methods

A total of 76 subjects aged 65–90 years were recruited from Gachon University Gil Medical Center, Incheon, Korea, including 26 healthy age-matched control subjects and 50 cognitive impairment confirmed according to the criteria described in our previous report [37]. Briefly, individuals with subjective cognitive complaints were first screened for cognitive impairment using a cut-off score of > 26 of 30 on the Mini-Mental State Examination (MMSE) [38]. Subjects scoring below 26 were subjected to detailed neuropsychological testing, including the Clinical Dementia Rating-Sum of Box (CDR-SOB; scores > 2.5) and Global Deterioration Scale (GDS; scores > 3), which are broadly accepted measures for dementia [39]. Patients with comorbidities were excluded. Patients were diagnosed according to American Psychiatric Association DSM-IV criteria. All clinical tests were performed by investigators blinded to the subjects’ genetic status; however, the blinded condition could not realistically be maintained for overtly demented subjects. Table 1 summarizes the clinical and demographic characteristics of the study population. The study was conducted according to the guidelines of the Ethics Committee of Gachon University Gil Medical Center and with the approval of the institutional review board of Gachon University Gil Medical Center (GAIRB2013-264, 23 October 2013, GCIRB2016-015, 21 January 2016). All the subjects provided written informed consent before participating via self-referral or referral from a family member.

### 4.2. Serum Separation

Ten milliliters (mL) of blood was collected from each participant by vein puncture into sterile vacutainers under strict aseptic conditions. The samples were kept at room temperature (RT) for 30–40 min to clot, and then centrifuged for 20 min at 1000× *g* to separate the serum. Serum was collected carefully, and a protease inhibitor cocktail (535140; EMD Biosciences, Inc., Darmstadt, Germany) and phosphatase inhibitor cocktail (P5726 and P0044; Sigma-Aldrich, Inc., St. Louis, Missouri, USA) were added. Serum samples were aliquoted and immediately stored at −80 °C until further analysis. Aliquots were thawed on the day of analysis.

### 4.3. Animals

JNPL3 mice expressing mutant human tau mutation (MAPT) were obtained from Taconic Farms (Germantown, NY, USA). All mice were genotyped by polymerase chain reaction analysis of tail DNA. Four- and 15-month-old WT and JNPL3 mice were used in this study. All animal procedures were approved by the Institutional Animal Care and Use Committee of the Lee Gil Ya Cancer and Diabetes Institute, Gachon University (IACUC No. LCDI-2019-0114, 24 June 2019, LCDI-2020-0033, 4 June 2020).

### 4.4. Isolation of NEX from Serum

From each patient group, 16 serum samples were selected randomly for NEX isolation. Briefly, 0.5 mL of serum was mixed with 252 µL of ExoQuick EX precipitation solution (EXOQ5A-1; System Biosciences, Inc., Palo Alto, CA, USA) and incubated for 1 h at 4 °C. After centrifugation at 1500× *g* for 30 min at 4 °C, the pellet was resuspended in 250 µL of calcium- and magnesium-free Dulbecco’s balanced salt solution (DPBS; Thermo Fisher Scientific, Waltham, MA, USA) with inhibitor cocktails.

The NEX fraction was then enriched according to a previous report [26]. Each sample was mixed with 100 µL of 3% bovine serum albumin (BSA) and incubated for 1 h at 4 °C, followed by the addition of 1 mg rabbit anti-human CD171/NCAM-L1 (L1 cell adhesion molecule [L1CAM]) biotin-conjugated antibody (bs-1996R-Biotin; Bioss Antibodies Inc., Woburn, MA, USA) and 25 µL streptavidin-agarose resin (53116, Thermo Fisher Scientific) plus 50 µL of 3% BSA. After centrifugation at 200× *g* for 10 min at 4 °C and removal of the supernatant, the pellet was resuspended in 50 µL of 0.05 M glycine-HCl (pH 3.0) by vortexing for 10 s. The suspension was then mixed with 0.45 mL of M-PER mammalian protein extraction reagent (78501, Thermo Fisher Scientific) and incubated for 10 min at 37 °C with vortex mixing. Extracted proteins were immediately stored at −80 °C until further analysis.

### 4.5. Isolation of TEX from Brain Tissue

Exosomes were extracted from brain tissue according to the methods of Perez-Gonzalez and colleagues [27] with minor modifications. Frozen (−80 °C) mouse brain (one hemisphere) was chopped in trypsin solution prewarmed to 37 °C at 0.1 g/mL. The tissue was then transferred to 15 mL tubes containing 1 × trypsin (Gibco BRL., Thermo Fisher Scientific) and incubated for 20 min at 37 °C with gentle shaking. Ice-cold DMEM (with protease inhibitor (PI), Gibco BRL) was added to stop digestion. The suspension was then mixed by being gently pipetted twice, filtered through 40 μm mesh, and centrifuged at 300 × *g* for 10 min at 4 °C to remove brain cells and tissue. The supernatant was transferred to a fresh tube and centrifuged at 2000 × *g* for 10 min at 4 °C. The second supernatant was then centrifuged at 10,000 × *g* for 30 min at 4 °C and sequentially filtered with 0.45 μm filter and 0.2 μm filter. Each filtrate was diluted with ice-cold phosphate-buffered saline (PBS) and centrifuged 3 times, each time at 100,000 × *g* for 70 min at 4 °C using an ultracentrifuge (SW28, Beckman, Miami, FL, USA) to pellet the vesicles. The final extracellular vesicle pellet was resuspended in 0.95 M sucrose solution, and centrifuged through a 6-layer sucrose gradient (0.25 M, 0.6 M, 0.95 M, 1.3 M, 1.65 M, and 2 M sucrose solution in 20 mM HEPES) at 200,000× *g* for 16 h at 4 °C using an ultracentrifuge (SW41, Beckman). Each fraction was diluted with ice-cold PBS and centrifuged at 100,000 × *g* (average) for 1 h at 4 °C (SW41, Beckman) to pellet the vesicles. The supernatant was discarded and pellets were collected in ice-cold PBS (with PI). The vesicles in fractions 1–3 were then characterized by a suite of techniques including transmission electron microscopy to confirm enrichment of exosomes.

### 4.6. Electron Microscopy

Analysis of exosomes by TEM was conducted with the support of the Brain Research Core Facilities at the Korea Brain Research Institute (KBRI, Daegu, Korea). Glow discharge Formvar-carbon coated grids were prepared using the PELCO easiBlow Glow Discharge system (Ted Pella Inc., Redding, CA, USA). Grids were glow discharged for 30 s at 15 mA. Exosome pellets were suspended in 0.15 M cacodylate buffer (pH 7.4), applied onto glow discharged Formvar-carbon coated EM grids (Ted Pella Inc.), and left for 1 min in the air to allow the membranes to absorb on the surface. The excess sample liquid was then blotted off using filter paper (3M, Maplewood, MN, USA). The grid surface was floated on the surface of a 4% uranyl acetate staining solution (EMS-CHEMIE, Eftec North America, Taylor, MI, USA) for 1 min and then the excess solution was blotted off using 3M filter paper. The grid surface was then floated on the surface of a water droplet, and the water blotted off with filter paper. This latter procedure was repeated 5 times, and the grid was then dried in the air. Grids were then examined using a Tecnai G2 transmission electron microscope (Thermo Fisher Scientific). Vesicle size was measured using ImageJ between groups, we measured the density of the band using ImageJ (National Institutes of Health [NIH], Bethesda, Maryland, USA, https://imagej.nih.gov/ij/, 1997-2016).

### 4.7. Nanoparticle Tracking Analysis

Exosome pellets were resuspended in 60 μL PBS. Sucrose gradient fractions SN_0,_ SN_Δ1,_ and SN_Δ2_ were concentrated in Amicon^®^ Ultra-4 10 kDa nominal molecular weight centrifugal filter units to a final volume of 60 μL and a 10 μL of each prepared fraction diluted to 1:100 in PBS. Pellets obtained from 2 mL of media with Exo-quick were resuspended in 1 mL of PBS for nanoparticle tracking analysis (NTA) (particle concentrations were corrected for this concentration factor). Samples were analyzed in the range of 3–15 × 10^8^/mL by NTA using the NanoSight NS500 (Malvern Panalytical, Worcestershire, UK) equipped with a 405 nm laser. Videos were acquired and analyzed using the accompanying NTA software (version 3.1, Malvern Panalytical). The number of vesicles in each sample was presented as particles/mL media (mean  ±  S.D., *n*  =  6).

### 4.8. Measurements of t-tau and p-tau Protein Levels

Serum concentrations of t-tau, p-tau, and amyloid-beta (Aβ_42_) were detected using the following ELISA kits: Human tau protein ELISA kit, human phospho-tau (S202, T205) protein ELISA kit, human phosphor-tau (T181) protein ELISA kit, human phosphor-tau (S231) protein ELISA kit, and human Aβ42 ultrasensitive ELISA kit (Table 2). We used 50 µL of the sample according to the manufacturer’s instructions and diluted it 1:5 if necessary.

Tau protein levels in NEX were quantified by ELISA kits, and number of exosomes in each NEX sample was quantified by ExoELISA for the CD63 antigen (EXOEL-CD63; System Biosciences, Inc., Palo Alto, CA, USA) according to the manufacturer’s instructions. The mean value for all CD63 determinations in each assay group was set to 1.00, and the relative values for each sample were used to normalize t-tau and p-tau protein levels. The samples and standards were measured in duplicate, and the means of the duplicates were used for the statistical analyses.

### 4.9. Western Blot Analysis

We used Western blot to confirm the changes in t-tau and p-tau protein levels in NEX. From each group, 9 NEX samples were selected randomly for the Western blot experiment. For easy analysis, total protein in 20 μL of each sample was separated by sodium dodecyl sulfate-polyacrylamide gel electrophoresis (SDS-PAGE) and transferred to polyvinylidene difluoride (PVDF) membranes (Merck Millipore, Darmstadt, Germany). Membranes were blocked with 5% nonfat dry milk prepared in Tris-buffered saline (TBS) (10 mM Tris pH 7.5, 150 mM NaCl) for 1 h at RT and then incubated at 4 °C overnight in the following primary antibodies diluted with TBS: primary rabbit anti-human CD63 IgG and primary rabbit anti-human TSG101 IgG for EX, goat anti-human NCAM-L1 IgG for NEX, goat anti-human tau (C-17) for t-tau, mouse anti-human phospho-PHF-tau pSer202 + Thr205 antibody (AT8), mouse anti-human phospho-Tau (Thr181) monoclonal antibody (AT270), and mouse anti-human phospho-tau (Thr231) monoclonal antibody (AT180) for p-tau (Table 2). After washing with TBS-T (20 mM Tris pH 7.5, 500 mM NaCl, 0.05% Tween 20), blotted membranes were incubated with horseradish peroxidase (HRP)-conjugated secondary antibodies (goat anti-rabbit IgG, goat anti-mouse IgG, or donkey anti-goat IgG at RT for 1 h (Table 2). After washing with TBS-T, bands were visualized by an enhanced chemiluminescence system (Thermo Fisher Scientific). Band densities were measured using ImageJ to estimate protein expression. For the comparison of NCAM-L1 expression among samples, results were first normalized to CD63 expression. For comparison of tau protein expression among groups, results were normalized to NCAM-L1 expression. All results were then expressed fold change relative to the AMC group.

### 4.10. Immunofluorescence

Mice were perfused transcardially with saline containing heparin for immunohistochemical analysis. Brains were isolated, fixed in 4% paraformaldehyde at 4 °C for 24 h, and incubated in 30% sucrose solution at 4 °C for 72 h. Frozen blocks of brain tissue were cut into 30 μm-thick coronal slices using a cryostat (Cryotome, Thermo Electron Corporation, Waltham, MA, USA), and these were stored at 4 °C in cryoprotectant solution (ethylene 30% and glycerol 30% in PBS). Brain slices were washed 3 times in PBS containing 0.2% Triton X-100, incubated in a blocking solution (0.5% BSA and 3% normal horse serum in 0.4% PBS with Tween 20) at RT for 1 h, and then incubated with mouse anti-human phospho-PHF-tau pSer202 + Thr205 antibody (AT8) (1:100, Thermo Fisher Scientific) at 4 °C. Labeled sections were washed 3 times, incubated with a fluorescent dye-conjugated secondary antibody (Alexa fluor 555 donkey anti-mouse, 1:300, Invitrogen, Thermo Fisher Scientific) at RT for 1 h, washed 3 times, and mounted onto slides using Antifade Mounting Medium with DAPI (Vector Laboratories, Burlingame, CA, USA). Specimens were examined under a Zeiss AxioImager Z1 microscope. Images were captured using an Axiocam HRC camera (Carl-Zeiss Microscopy GmbH, Jena, Germany) and analyzed using ImageJ v1.4.3.67.

### 4.11. Statistical Analysis

Results were expressed as mean ± standard error of the mean. Normality was assessed using the Kolmogorov–Smirnov test. If the samples were normally distributed, differences between groups were determined by unpaired t-test or one-way analysis of variance (ANOVA) followed by Tukey’s multiple comparison tests. If the samples were non-normally distributed, differences were determined by the Mann–Whitney test or Kruskal–Wallis test followed by Dunn’s multiple comparison tests. Correlations were assessed using the nonparametric Spearman’s rank correlation test. Graphs show regression lines with 95% confidence intervals. Receiver operating characteristics (ROC) analyses were conducted under the nonparametric distribution assumption for the standard error of area to determine the capacities of the measurements to discriminate AD from AMCs. All statistical analyses were performed using Graph Pad Prism 8 (Version 8.4.2, GraphPad Software Inc., San Diego, CA, USA). A *p* < 0.05 (two-tailed) was accepted as statistically significant for all tests.

## Figures and Tables

**Table 1:** Demographic and clinical parameters of studied groups.

VARIABLES	AMC	MCI	Mild-AD
**Number of subjects**	26	30	20
**Female (F)/Male (M)**	9 (F)/17 (M)	18 (F)/12 (M)	17 (F)/3 (M)
**Age (years)** **(mean ± SEM)**	73.92 ± 0.88	75.13 ± 0.99	76.55 ± 1.33
**MMSE** **(mean ± SEM)**	27.69 ± 0.16	23.17 ± 0.20 ***	16.55 ± 0.52 ****^,##^
**CDR-SOB** **(mean ± SEM)**	0.75 ± 0.07	2.55 ± 0.03 ****	4.48 ± 0.28 ****^,#^
**GDS** **(mean ± SEM)**	2.00 ± 0.00	3.00 ± 0.00 ****	3.75 ± 0.14 ****

AMC, age-matched control; MCI, mild cognitive impairment; Mild-AD, mild dementia in Alzheimer’s disease; MMSE, Mini-Mental Status Examination; CDR-SOB, Clinical Dementia Rating-Sum of Box; GDS, Global Deterioration Scale. *** *p* < 0.001 and **** *p* < 0.0001 compared with the AMC subjects, ^#^ *p* < 0.05 and ^##^ *p* < 0.01 compared with the MCI subjects, using One-way ANOVA and Dunn’s Multiple comparison test.

**Table 2:** Antibodies and ELISA kits used in this study.

Target	Species	Dilution	Company (Catalog)
***For Western blot analysis***			
Anti-CD63 IgG	Rabbit	1:1000	System Biosciences (EXOAB-CD63A-1)
Anti-TSG101 IgG	Rabbit	1:1000	Abcam (AB125011)
Anti-NCAM-L1 IgG	Goat	1:1000	Santa Cruz Biotechnology (SC-1508)
Anti-Total tau (C-17)	Goat	1:500	Santa Cruz Biotechnology (SC-1995)
Anti-Phospho tau (S202, T205) (AT8)	Mouse	1:500	Thermo Fisher Scientific (MN1020)
Anti-Phospho tau (T181) (AT270)	Mouse	1:500	Thermo Fisher Scientific (MN1050)
Anti-Phospho tau (T231) (AT180)	Mouse	1:500	Thermo Fisher Scientific (MN1040)
Anti-mouse IgG HRP		1:3000	Biorad (170-6516)
Anti-rabbit IgG HRP		1:5000	Biorad (170-6515)
Anti-goat IgG HRP		1:5000	Santa cruz (SC-2020)
***For ELISA***			
Total tau			MyBioSource (MBS022635)
Phospho tau (S202, T205)			MyBioSource (MBS013458)
Phospho tau (T181)			MyBioSource (KHB00631)
Phospho tau (S231)			MyBioSource (KHB8051)
Amyloid bets1-42			Thermo Fisher Scientific (KHB3544)
CD63			System Biosciences (EXOEL-CD63)
***For immunofluorescence staining***			
Anti-Phospho tau (S202, T205)	Mouse	1:100	Thermo Fisher Scientific (MN1020)
Alexa Fluor 555 Donkey anti mouse IgG		1:300	Invitrogen (A-21422)

## Supplementary Materials

Supplementary materials can be found at https://www.mdpi.com/1422-0067/21/14/5007/s1.

## Abbreviations

AD	Alzheimer’s disease
Aβ	Amyloid-beta
AMC	age-matched control
APP	Amyloid precursor protein
CDR-SOB	Clinical Dementia Rating-Sum of Box
CSF	cerebrospinal fluid
DAB	3,3′–diaminobenzidine
DAPI	4′6-diamidino-2-phenylindole
ELISA	enzyme-linked immunosorbent assays
GDS	Global Deterioration Scale
IHC	Immunohistochemistry
MCI	Mild cognitive impairment
MMSE	Mini-Mental State Examination
NEX	neuronal-derived exosomes
NFTs	Neurofibrillary tangles
NINCDS-ADRDA	National Institute of Neurological and Communicative Disorders and Stroke-Alzheimer’s Disease and Related Disorders Association
PBS	Phosphate-buffered saline
PFA	Paraformaldehyde
p-tau	phosphorylated tau
TBS	Tris-buffered saline
°C	Celsius
TEM	Transmissin electron microscopy
t-tau	total tau
