# Supplementary material for: Serum Tau Proteins as Potential Biomarkers for the Assessment of Alzheimer’s Disease Progression
Source: Int J Mol Sci. 2020 Jul 15;21(14):5007. doi: 10.3390/ijms21145007 (PMC7404390; doi:10.3390/ijms21145007)
Supplement: Supplementary file 1 [file ijms-21-05007-s001.pdf]

Serum Tau Proteins as Potential Biomarkers for the Assessment of Alzheimer's Disease Progression

Eunjoo Nam, Yeong-Bae Lee, Cheil-Moon and Keun -A Chang

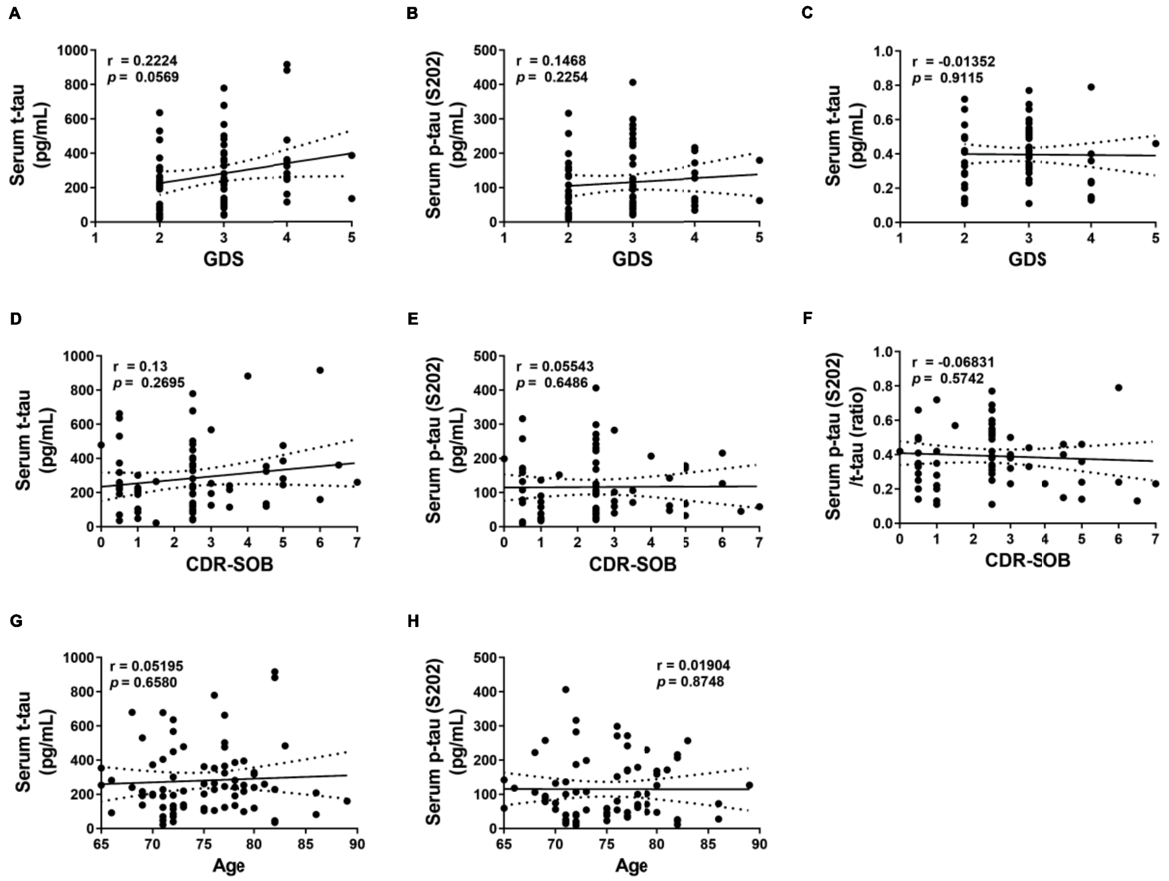

Figure S1. Total tau protein levels in human serum are correlated with Global Deterioration Scale (GDS) scores but not with age. Correlations of serum **A**) t-tau, **B**) p-tau (S202), and **C**) p-tau (S202)/t-tau with GDS were assessed using the nonparametric Spearman's rank correlation test. Correlations of serum **D**) t-tau, **E**) p-tau (S202), and **F**) p-tau (S202)/t-tau with CDR-SOB were assessed using the nonparametric Spearman's rank correlation test. Graphs show regression lines with 95% confidence intervals. Serum t-tau levels were correlated with GDS scores. Correlation of serum **G**) t-tau and **H**) p-tau (S202) with age were assessed using the nonparametric Spearman's rank correlation test.

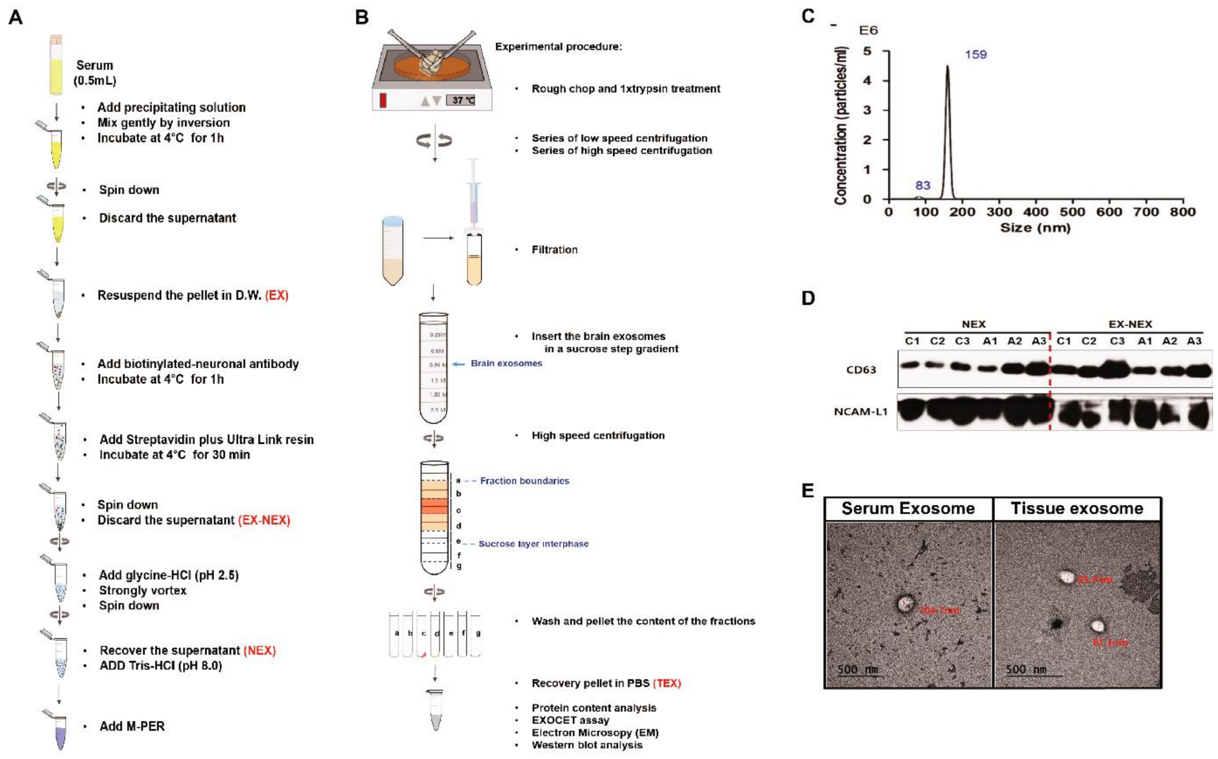

Figure S2. Enrichment and characterization of exosomes. **A**)Workflow of the neuronal cell-derived exosome (NEX) enrichment protocol. **B**) Workflow of the tissue exosome (tEX) enrichment protocol. **C**) Exosome (EX) particle size was measured using NanoSight. **D**) NEX enrichment was validated by Western blotting for the exosome marker CD63 and neuronal marker NCAML1. NEX, neuronal cell derived exosome-enriched fraction; EX-NEX, exosomes except NEX fraction. **E**) TEM image of exosomes.

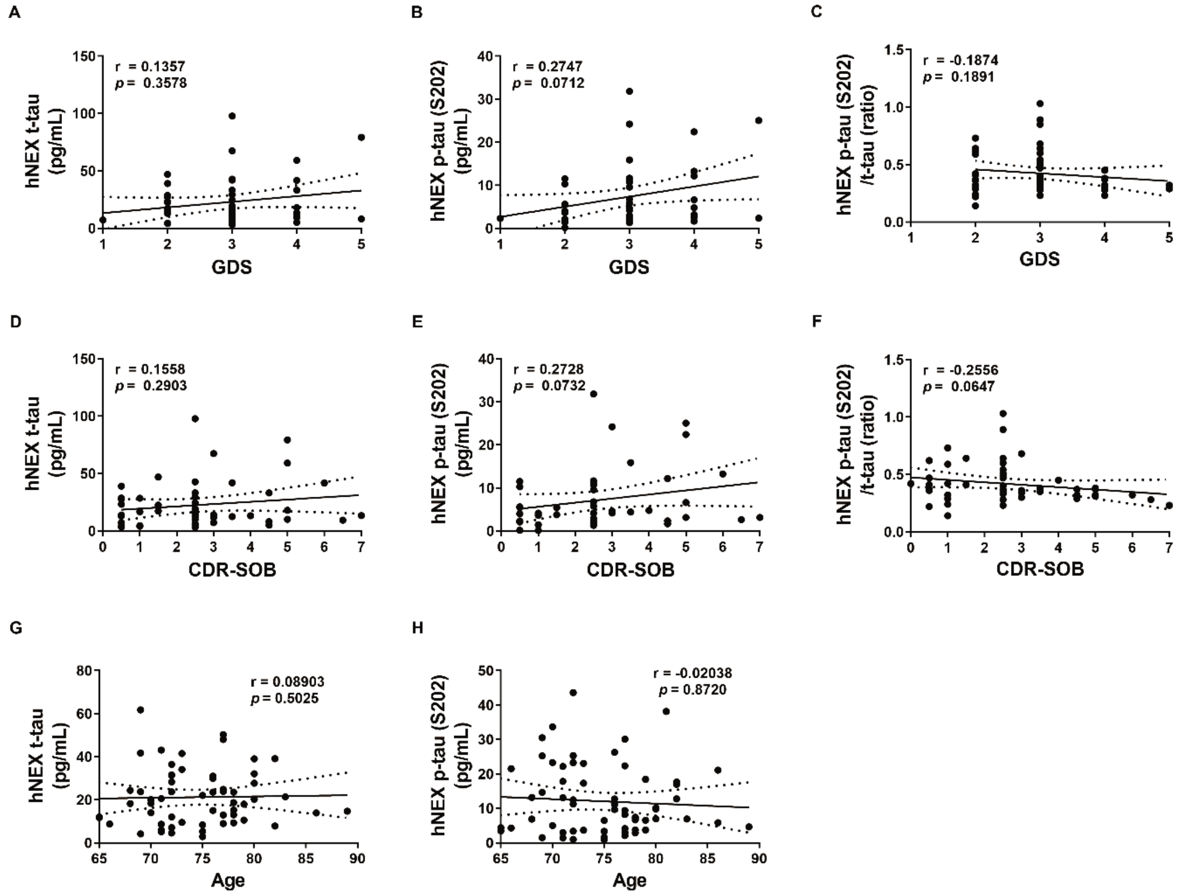

Figure S3. Phosphorylated tau protein levels in human neuronal cell-derived exosomes are correlated with GDS and Clinical Dementia Rating (CDR)-Sum of Boxes (SOB) scores but not with age. Correlations of hNEX **A**) t-tau, **B**) p-tau (S202), and **C**) p-tau (S202)/t-tau with GDS scores were assessed using the nonparametric Spearman's rank correlation test. Correlation of hNEX **D**) t-tau, **E**) p-tau (S202), and **F**) p-tau (S202)/t-tau with CDR-SOB scores were assessed using the nonparametric Spearman's rank correlation test. Graphs show regression lines with 95% confidence intervals. hNEX p-tau (S202) levels were correlated with GDS and CDR-SOB scores. The correlations of hNEX C) t-tau and **D**) p-tau (S202) with age were assessed using the nonparametric Spearman's rank correlation test. There were no correlations between tau proteins and age.

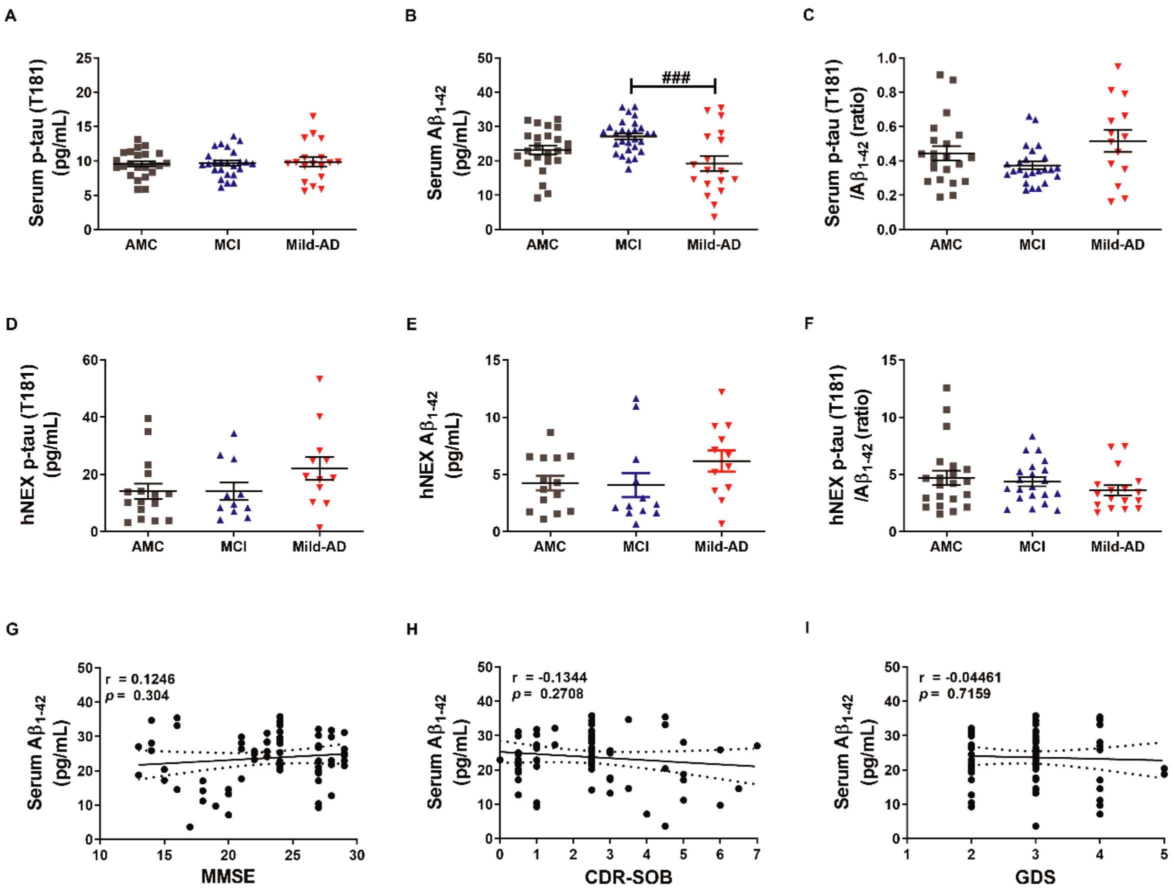

Figure S4. Phosphorylated tau (T181) and amyloid beta levels are not elevated in serum and neuronal cell-derived exosomes of Alzheimer's disease patients. **A**) Phosphorylated tau (p-tau (T181)), **B**) amyloid beta ($A\beta_{1-42}$), and **C**) p-tau (T181)/$A\beta_{1-42}$ ratio in human serum were quantified using ELISA. Serum $A\beta_{1-42}$ levels were significantly lower in the Mild-AD group than the MCI group. **D**) p-tau (T181), **E**) $A\beta_{1-42}$ and **F**) p-tau (T181)/$A\beta_{1-42}$ in hNEX were quantified using ELISA. There were no differences between groups. All data were shown as means ± SEM. $^{\#\#\#}p < 0.001$ compared to the MCI group by one-way ANOVA and Bonferroni's multiple comparison test. The correlations of serum $A\beta_{1-42}$ with **G**) MMSE, **H**) GDS, and C) CDR-SOB were assessed using the nonparametric Spearman's rank correlation test. Graphs show regression lines with 95% confidence intervals. There was no correlation between serum $A\beta_{1-42}$ and cognition test scores.

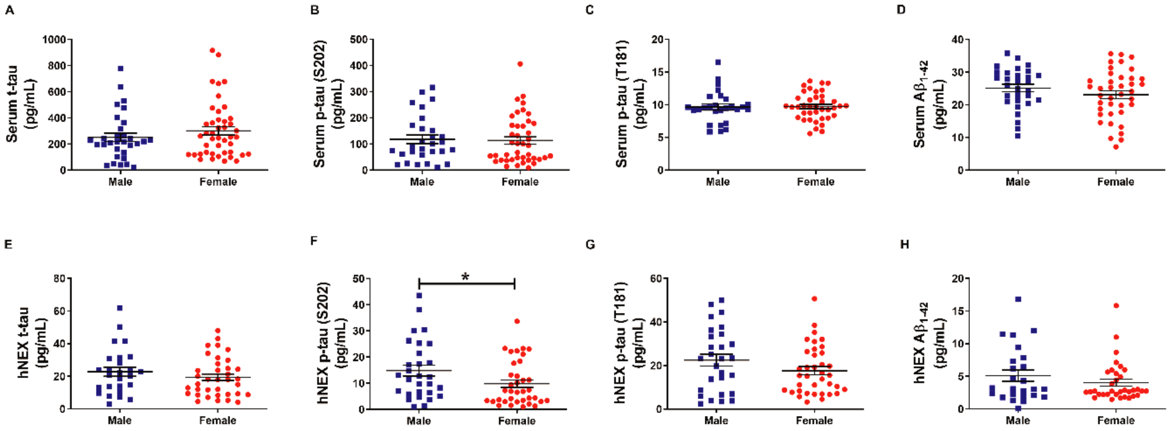

Figure S5. Phosphorylated tau (S202) protein levels in human neuronal cell-derived exosomes are lower in female AD patients. Comparison of serum **A**) t-tau, **B**) p-tau (S202), **C**) p-tau (T181) and **D**) Aβ_{1-42} between male and female AD patients. Comparisons of hNEX, **E**) t-tau, **F**) p-tau (S202), **G**) p-tau (T181), and **H**) Aβ1-42 between male and female AD patients. hNEX p-tau (S202) levels were lower in female patients than male patients. All data were shown as means ± SEM. * $p < 0.05$ compared to males by Mann–Whitney test.

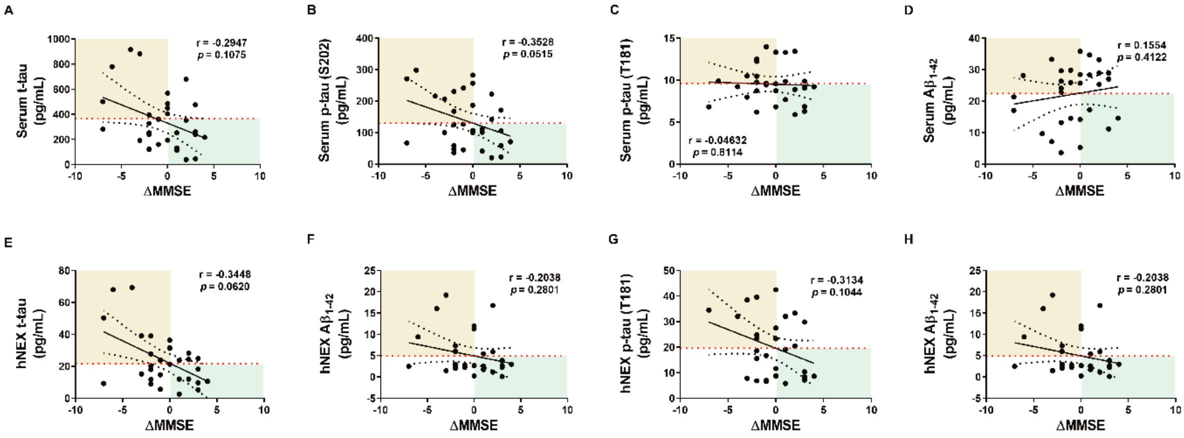

Figure S6. Phosphorylated tau protein levels in human serum and neuronal cell-derived exosomes are correlated with ΔMMSE scores. Correlations of serum **A**) t-tau, **B**) p-tau (S202), **C**) p-tau (T181), and **D**) $A\beta_{1-42}$ with ΔMMSE were assessed using the nonparametric Spearman's rank correlation test. Correlations of hNEX **A**) t-tau, **B**) p-tau (S202), **C**) p-tau (T181), and **D**) $A\beta_{1-42}$ with ΔMMSE were assessed using the nonparametric Spearman's rank correlation test. Serum p-tau (S202) levels were correlated with ΔMMSE scores.

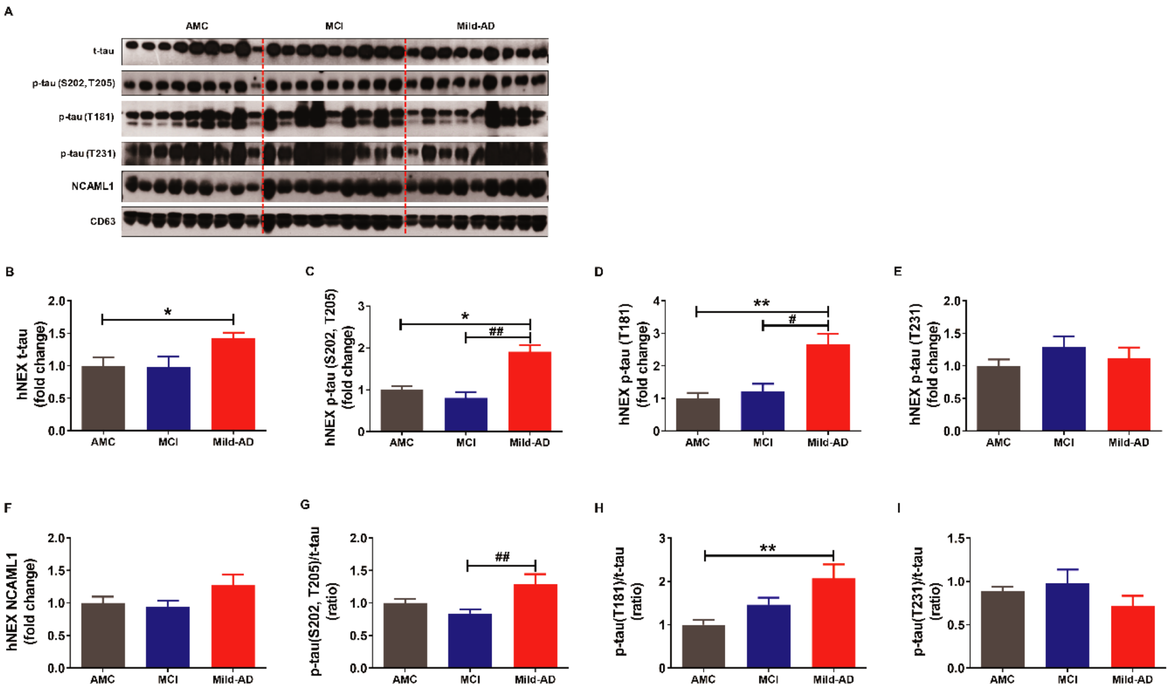

Figure S7. Total tau and phosphorylated tau protein expression levels in neuronal cell-derived exosomes increase with Alzheimer's disease severity. Total tau and phosphorylated tau protein expression levels in hNEX were validated by Western blot. **A**) Representative Western blot. Relative expression levels of **B**) t-tau, **C**) hyper-phosphorylated tau (p-tau (S202, T205)), **D**) p-tau (T181), **E**) p-tau (T231), and F) NCAML1 as well as **G**) p-tau (S202, T205)/t-tau ratio, H) p-tau (T181)/t-tau ratio, and I) p-tau (T231)/t-tau ratio in hNEX. hNEX t-tau and p-tau (T181)/t-tau were higher in the Mild-AD group than the AMC group. hNEX p-tau (S202, T205) was higher in the Mild-AD group than the AMC and MCI groups. All data were shown as means ± SEM, and each experiment was repeated five times ($n = 9$ per group). $^{*}p < 0.05$ and $^{**}p < 0.01$ compared to the AMC group, and $^{\#}p < 0.05$ and $^{\#\#}p < 0.01$ compared to the MCI group by one-way ANOVA and Bonferroni's multiple comparison test.

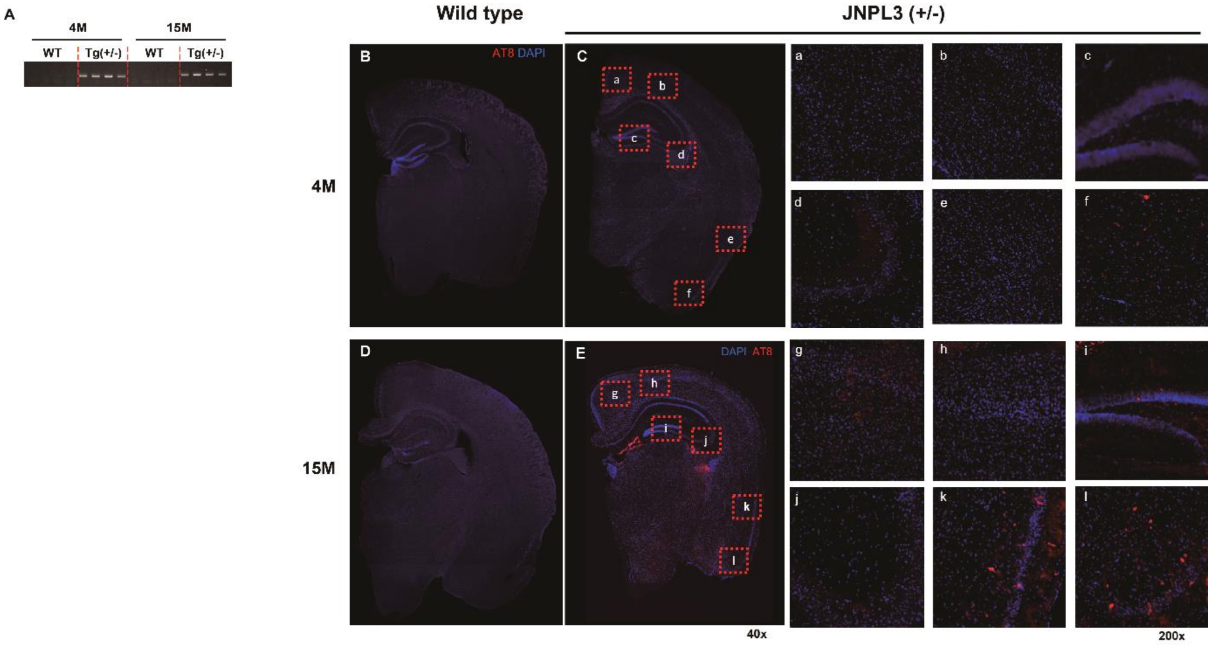

Figure S8. Characterization of JNPL3 mice. **A**) Representative genotyping. 4-month-old wild type mice (4M-WT, n = 14), 4-month-old JNPL3 mice (4M-Tg, n = 14), 15-month-old wild type mice (15M-WT, n = 17) and 15-month-old JNPL3 mice (15M-Tg, n = 19). B-l) Expression of the hyper-phosphorylated tau marker AT8 was evaluated using immunohistochemistry (n = 3 per group). **B**) 4M-WT, **C**) 4M-Tg, **D**) 15M-WT, and **E**) 15M-Tg at low magnification (40×). High magnification (200×) images of **a**-**c**) hippocampus and **d**-**f**) cortex in 4M-Tg mice, and **g**-**i**) hippocampus and **j**-**l**) cortex in 15M-Tg mice.
